# SARS-CoV-2 Footprints in the Placenta: What We Know after Three Years of the Pandemic

**DOI:** 10.3390/jpm13040699

**Published:** 2023-04-21

**Authors:** Valentina Tosto, Arun Meyyazhagan, Malek Alqasem, Valentina Tsibizova, Gian Carlo Di Renzo

**Affiliations:** 1 Centre of Perinatal and Reproductive Medicine, University of Perugia, 06121 Perugia, Italy; tosto.valentina@libero.it (V.T.); arun47biotech@gmail.com (A.M.); 2 Perinatology Research Branch, Wayne State University, Detroit, MI 48202, USA; 3 Department of Life Sciences, CHRIST (Deemed to be University), Bengaluru 560029, India; 4 Department of Obstetrics and Gynecology, Faculty of Medicine, Mutah University, Al-Karak 61710, Jordan; malikmutah85@gmail.com; 5 PREIS International School, 50122 Firenze, Italy; tsibizova.v@gmail.com; 6 Department of Obstetrics and Gynecology, IM Sechenov First State University, 19c1, Moscow 119991, Russia; 7 Almazov National Medical Research Centre, St Petersburg 197341, Russia

**Keywords:** COVID-19, SARS-CoV-2, placental pathology, entry factors, vertical transmission, obstetric outcomes, perinatal outcomes

## Abstract

As the COVID-19 pandemic continues into its third year, there is accumulating evidence on the consequences of maternal infection. Emerging data indicate increased obstetrics risks, including maternal complications, preterm births, impaired intrauterine fetal growth, hypertensive disorders, stillbirth, gestational diabetes, and a risk of developmental defects in neonates. Overall, controversial concerns still exist regarding the potential for vertical transmission. Histopathological examination of the placenta can represent a useful instrument for investigation and can contribute significant information regarding the possible immunohistopathological mechanisms involved in developing unfavorable perinatal outcomes. Based on current evidence, SARS-CoV-2 infection can affect placental tissue by inducing several specific changes. The level of placental involvement is considered one of the determining factors for unfavorable outcomes during pregnancy due to inflammation and vascular injuries contributing to complex cascade immunological and biological events; however, available evidence does not indicate a strong and absolute correlation between maternal infection, placental lesions, and obstetric outcomes. As existing studies are still limited, we further explore the placenta at three different levels, using histology, immunohistochemistry, and molecular genetics to understand the epidemiological and virological changes observed in the ongoing pandemic.

## 1. Introduction

Since its emergence in December 2019, the highly contagious 2019 novel coronavirus disease (COVID-19) has affected more than 759,408,703 people and killed more than 6,866,434 people around the globe. A total of 13,229,166,046 vaccine doses have been administered to date [1]. The infection, however, continues to spread in its third year with evolving virological and epidemiological changes along with a broad spectrum of clinical manifestations. Most severe acute respiratory syndrome coronavirus 2 (SARS-CoV-2)-positive patients are asymptomatic or manifest mild upper respiratory infection symptoms, which can occasionally progress into severe respiratory illness, multiorgan damage, organ failure, and even death [2,3].

As a susceptible group, pregnant women are at higher risk of hospitalization, admission to intensive care, mechanical ventilation, and early delivery with nearly the same mortality rates for both pregnant and non-pregnant women [4]. During pregnancy, SARS-CoV-2 is linked with a maternal inflammatory response in circulation and the interface between the mother and fetus. Elevated levels of IgM and IgG were observed in peripheral circulation, with IgG detected in neonatal cord blood as it crosses the placental barrier through the Fc receptor of the neonate [5]. Maternal comorbidities such as diabetes mellitus, advanced maternal age, gestational hypertension, and obesity can increase the severity of COVID-19 [6].

The current review focuses on the transmission of SARS-CoV-2 through the placenta, its mechanisms and responses towards the infection, and the cellular and molecular defensive role of the placenta.

## 2. Mode of Entry

Only a handful of viruses can cross the placental barrier and lead to birth defects and pregnancy complications. These viruses include zika virus, human cytomegalovirus, rubella, herpes, and possibly SARS-CoV2. SARS-CoV-2 enters host cells mainly via the angiotensin-converting enzyme 2 (ACE2) receptor [7]. While lung cells are the primary targets of this respiratory virus, causing acute respiratory distress syndrome, the virus can also affect other ACE2-expressing tissues, including those of the cardiovascular system [8]. Inevitably, the effects of SARS-CoV-2 infection on vulnerable populations such as pregnant women and their fetuses have caught worldwide attention. Overall, data collected since the beginning of the pandemic have shown heterogeneous results due to obvious limitations related to studying an unknown pathogen. Studies, including systematic reviews, found that pregnant women with SARS-CoV-2 infection had significantly higher odds of pre-eclampsia, preterm birth, stillbirth, and intensive care unit (ICU) admission compared to those without infection [9].

### Vertical Transmission of SARS-CoV-2 from Mother to Fetus

Transmission of a viral load from the infected mother to the fetus in the intrauterine, intrapartum, or postnatal period is defined as vertical transmission. This type of transmission generally occurs through a transplacental vector in utero, through a cervicovaginal avenue during delivery, or through close contact between the child and mother during the postnatal period. Testing for SARS-CoV-2 in placental samples from pregnant women showed a higher viral RNA load than that found in the amniotic fluid and vaginal secretions of cord blood, showing through systemic analysis that transplacental transmission is more likely than cervicovaginal transmission [10,11]. Transplacental transmission is facilitated by trans-membrane serine protease 2 (TMPRSS2) primed with the spike protein domain and permitting viral entry through ACE2 receptors seen in the placental trophoblast [12]. Based on latest studies, placental cells use human dipeptidylpeptidase-4 (DPP4), CD-14, and cathepsin-L (CTSL) mediators during pregnancy to permit the entry of SARS-CoV-2 [13]. The virion can infiltrate the placenta by infecting maternal immune cells (cell-to-cell transmission), or via transcytosis, as the trophoblast cells can transpose opsonized or free viral particles in an endosomal pattern. The presence of ischemia or inflammations will aid in transmigration of the virion more effectively to the fetal environment [14].

## 3. SARS-CoV-2 Consequences for the Placenta

The virion can negatively impact pregnancy after infection either through cell death (apoptosis, pyroptosis, novel ferroptosis, or necroptosis) or through excessive inflammation [15]. Recent studies have shown ferroptosis to be the main cause underlying multiple organ damage and failure in COVID-19-positive individuals. Most notably, lipid repairing capacity is hindered by disproportionate iron-dependent hydroxyperoxidation in cell membrane polyunsaturated fatty acids [16]. Secondly, the virion codes with open reading frames (ORF3a and 7b), yielding apoptosis through caspase-8 activation independent of BCL-2 expression and leading to compromised integrity and functioning of the placenta, ultimately, causing growth restrictions, pre-eclampsia, and early rupture [14].

**Figure 1:**
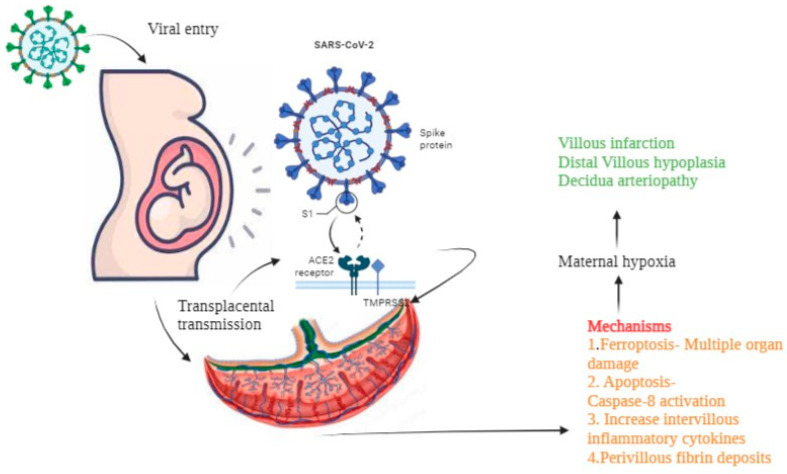
The influence of SAR-CoV-2 on placental development.

SARS-CoV-2 placentitis occurs due to histiocyte-dominant intervillous inflammatory cytokines and perivillous fibrin deposition causing massive inflammatory responses and elevating the risk of vertical transmission [17] (Figure 1). Maternal hypoxia following viral invasion can reduce blood flow in the placenta and manifest as maternal vascular malperfusion, causing villous infarction, hypoplasia of the distal villi, and arteriopathy in the decidua [14]. Histomorphological changes in the placenta to different variants of SARS-CoV-2, the rate of vertical transmission, the time between infection and delivery, and immunization status are some of the key areas awaiting further study.

## 4. SARS-CoV-2 Infection and Pregnancy Outcomes

Several unfavorable perinatal outcomes were reported in cases of elevated SARS-CoV-2 infections during gestation such as miscarriage, preterm premature rupture of the membranes (PPROM), low birth weight, impaired fetal growth (small for gestational age and intrauterine growth restriction), hypertensive disorders, gestational diabetes mellitus (GDM), lower fetal movements, fetal distress, higher cesarean rate, neonatal intensive care (NICU) admission, and neonatal death as the renin–angiotensin system (RAS) is downregulated, causing increased blood pressure and placental vascularization dysfunction [18]. The maternal and perinatal consequences of SARS-CoV-2 infections are gaining interest to determine the possible effects among youth and adults as “in utero” programming. Future investigations may provide answers to these questions through long-term follow-ups.

Interestingly, new insights from a few recent studies suggest that fetal brain tissues such as syncytiotrophoblasts and cytotrophoblasts could be affected by SARS-CoV-2. ACE2 and trans-membrane serine protease 2 (TMPRSS2) show low expression, but furin is highly expressed in the fetal brain. Thus, these molecules may play a role in the pathogenic infection of the fetal brain during the second and third trimesters of pregnancy [19]. It was also reported that SARS-CoV-2 infection may result in lower fetal movements seen as bilateral fronto-parieto-occipital cystic periventricular leukomalacia postnatally on day 25, suggesting that infection may cause damage to newborn brains [20].

The majority of newborns delivered by pregnant women infected with SARS-CoV-2 tested negative for the virus, but a few tested positive. In these cases, it is important to determine whether intrauterine transmission of SARS-COV-2 occurred and its development mechanisms. The rate of transmission through the placenta to the fetus reported in women with COVID-19 was reported to be low. The relative rarity of materno-fetal transmission may be attributable to several factors. The virus must first reach the placenta and cross it, and SARS-CoV-2 is known to have a very low level of viremia. Furthermore, as recently suggested by Gengler et al. [21], the level of receptor expression that aids in facilitating entry of the virus is very low in placental tissues; however, controversy exists in published studies regarding such levels.

## 5. Placental Defense Mechanism

Notably, placental examination can provide essential information on changes to the human placental structure and the mechanisms of maternal–fetal transmission [22] as well as the effects of organisms on the placenta due to viral infection, such as abnormal inflammatory responses, vascular changes, hemorrhagic lesions, and necrosis [23]. The placenta has a unique capacity to permit, prevent, or limit expansion of the virus and transmission to the fetus, acting simultaneously as both a friend and enemy [24,25,26].

It is well documented that the human placenta plays an essential role in modulating the immune responses in several viral infections [27]. Some viruses can cross the placental barrier and induce associated severe fetal malformations, such as having pathological, unfavorable perinatal, and/or long-term effects [28]. The risk of placental adverse outcomes may be due to malperfusion, thrombosis, and fibrin deposition within the placenta [29,30,31]. There is still a pressing need to understand the pathogenesis of SARS-CoV-2, promote disease prevention strategies, enhance diagnostics, review existing therapeutics and innovate new ones, and provide safe pipelines for the development of more effective vaccines, considering the dynamic viral and epidemiological changes related to this disease.

## 6. Route of SARS-CoV-2 during Pregnancy and Its Consequences

**Figure 2:**
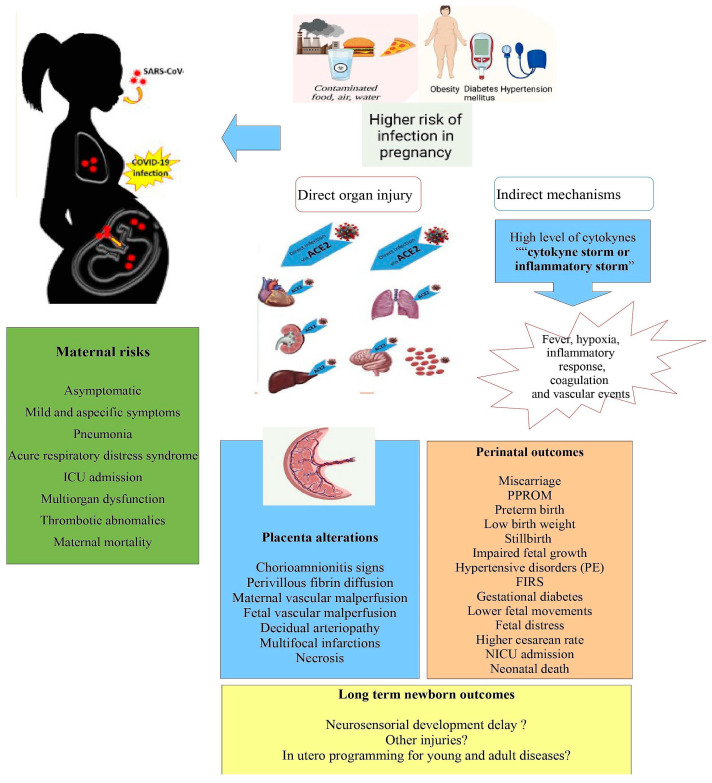
Schematic overview of factors and pathogenic mechanisms underlying the possible adverse maternal and feto-neonatal outcomes in SARS-CoV-2 pregnancies.

Placental pathological studies related to SARS-CoV-2 infection suggest significant changes. Notably, chronic alterations such as histiocytic intervillositis are often detected and involved in mother-to-child transmission with variable significance on clinically unfavorable perinatal outcomes in a few studies (Figure 2).

It seems contradictory that infected placentas comprise the majority of drastic morphological changes based on the severity of infection. In other cases, the morphology is similar to that of non-infected patients. A higher prevalence of necrotic trophoblasts was described in the villi of women requiring respiratory support, showing that the severity of the disease is associated with histopathological changes observed in the placenta after infection with SARS-CoV-2 [32]. Advanced methodologies and techniques of placental investigation are available and included in morphological and morphometrical analysis, such as immunohistochemistry studies, transcriptome sequencing (RNA-seq), real-time quantitative PCR (RT-qPCR), in situ hybridization, immunofluorescence techniques, and transmission electrode microscopes.

Several studies focused on evaluating SARS-CoV-2 entry factors. Entry factors are receptors and other molecules that can increase or reduce the permissiveness of the placenta towards SARS-CoV-2 entry. Few studies have investigated how the localization of SARS-CoV-2 receptors, proteases, and genes involved in coding proteins drive viral pathogenesis in the placenta, showing probable variability in each trimester of pregnancy [33].

Angiotensin-converting enzyme 2 (ACE2) receptor expression plays a key role in this infection, and two conditions seem necessary for transplacental transmission: (a) the virus must reach the placenta, and (b) the ACE2 receptor must be expressed in placental tissue. Regarding the first point, published data support the presence of SARS-CoV-2 in placental tissue [10,33,34,35,36]; however, regarding the second condition, the data remain conflicting [37,38]. By testing placental tissues at various gestational ages in both SARS-CoV-2-positive and -negative mothers, Gengler et al. confirmed that ACE expression is present consistently throughout pregnancy, regardless of SARS-CoV-2 status [19]. The expression of abnormal levels of trans-membrane serine protease 2 (TMPRSS2) and furin was also postulated as a primary factor that could further facilitate viral entry [39].

Essalmani et al. suggested that furin and TMPRSS2 act synergistically in viral entry and infectivity, supporting the combination of furin and TMPRSS2 inhibitors as potent antivirals against SARS-CoV-2 [40]. The relatively low transmission risk of SARS-CoV-2 to the feto-placental unit was recently attributed to a negligible expression of SARS-CoV-2 entry factors in the human placenta. Current epidemiological data suggest that the feto-placental unit is largely resistant to SARS-CoV-2 infection. Based on these observations, it is likely that SARS-CoV-2 enters the trophoblasts, albeit at a lower level than that of permissive lung epithelial cells. SARS-CoV-2 replication in the human placenta may be limited by the failure to activate post-entry pathways, such as endosomal escape or the lysosomal deacidification pathways [37].

Intriguingly, Shook et al. investigated whether placental defenses in SARS-CoV-2 maternal infection might be mediated by fetal sex. In particular, the authors investigated whether placental ACE2 and TMPRSS2 expression varied by fetal sex. Ultimately, no impact of fetal sex or maternal SARS-CoV-2 status on ACE2 was observed. TMPRSS2 expression was significantly correlated with ACE2 expression in males but not females [41]. The identification of a possible sexually dimorphic response to maternal SARS-CoV-2 infection in placental TMPRSS2 levels represents an important element for understanding whether and how fetal sex impacts the risk for transplacental transmission. Whether lower TMPRSS2 levels in the presence of maternal SARS-CoV-2 infection reflect a protective compensatory process in the male placenta, potentially triggered by activation of the maternal innate immune system in response to SARS-CoV-2 infection, warrants further study [41].

The presence of alternative receptors for SARS-CoV-2 entry into syncytiotrophoblast cells has also been suggested, including CD47, HLA G, CD 26, and CD56 expression [42,43]. A recent study by Dong et al. reported the differential expression of both ACE2 and CD147 in a small cohort of pregnant women associated with SARS-CoV-2 placental infection compared to non-infected pregnant women, with no direct evidence of viral transmission to the newborns [42]. Furthermore, peculiar escape mechanisms sometimes exhibited by the SARS-CoV-2 virus corresponded to the induction of the immunotolerogenic molecule human leukocyte antigen (HLA)-G. HLA-G placental expression during pregnancy is characterized by peculiar changes, with high levels of the molecules in the first trimester, which then reduce as they approach birth in order to promote the typical inflammatory environment needed to trigger delivery [44].

Most recently, Schiuma et al. [43] reported modifications in the immune environment, including an increase in the immune-tolerogenic molecule HLA-G that can act as immune-escape mechanism for SARS-CoV-2 and a decrease in CD56-expressing immune cells. A decrease in CD56 expression induces a cytotoxic phenotype that might alter the immune tolerogenic status at the fetal–maternal interface [42]. Other studies focused on the expression of molecules and receptors in relation to infection [45,46,47,48,49]. The majority of published data agree that asymptomatic or mildly symptomatic SARS-CoV-2-positive pregnant women with otherwise uncomplicated pregnancies present placental injury at a microscopic level but without observable poor pregnancy outcomes. This does not obviate the increased risk of unfavorable conditions for both the mother and the fetus, as SARS-CoV-2 infection remains a risk factor for systemic pro-inflammatory, thrombotic, and microvascular injury syndrome in the context of a complex vicious cycle.

Recently, Celik et al. [49] indicated that disease severity is associated with ischemic placental pathology, which may result in adverse pregnancy outcomes such as preterm birth and intrauterine growth restriction [30]. The pathophysiological mechanisms behind the development of lesions in the placentas of some mothers infected with SARS-CoV-2 remain to be elucidated; however, the synergistic effect of immunological dysregulation induced by the virus and underlying or acquired thrombophilia in the mother or fetus may trigger the relevant pathological pathways.

Gychka et al. [50] provided a morphometric and immunohistochemistry analysis of placental tissue. Interestingly, the authors observed significant remodeling of vessels in the placentas of SARS-CoV-2-positive women. Morphometric analysis of placental arterial wall thickness showed that the median value for SARS-CoV-2 patients was ~30 μm, while that for controls was ~15 μm, indicating that the placental arterial walls were twice as thick in SARS-CoV-2 patients than those in women without SARS-CoV-2. The placental arterial lumen area was found to be significantly smaller (5-fold) in SARS-CoV-2 patients than in the controls (*p* < 0.01). In addition, immunohistochemistry using the smooth muscle cell marker α-smooth muscle actin clearly indicated a dramatic increase in smooth muscle mass in the placental arteries of SARS-CoV-2 patients, with quantitatively thickened placental vessels [50]. Another recent case–control study reported significant pathological alterations in the placenta and umbilical cord [51].

Using immunohistochemistry, Perna et al. [52] studied the expression of CD34 and podoplanin (PDPN) as markers of vasculogenesis in uncomplicated pregnancies with SARS-CoV-2 infection during the first, second, or third trimester of gestation. Results showed PDPN expression around the villous stroma as a plexiform network around the villous nuclei of fetal vessels; significant down-regulation was also observed in the villous stroma of women infected during the third trimester. CD34 showed no changes in expression levels [52].

In general, case series reports consistently describe a considerable proportion of placentas with histopathological anomalies, including fibrosis, necrosis, and vascular injuries [53]; nevertheless, the lower rate of vertical transmission indicates the potent role of the placental barrier in inhibiting viral transmission to the fetus, thereby limiting unfavorable perinatal outcomes. Less than about 20% of placental tissues were found to be PCR positive, showing a significant grade of resistance to SARS-CoV-2 infection. Takada K et al. directly demonstrated inefficient viral replication in a SARS-CoV-2-infected placenta [54].

Table 1 provides an overview of the main entry factors and other molecules investigated for their likely involvement in SARS-CoV-2’s capacity to infect placental tissue [37,38,39,40,41,42,43,44,45,46,47,48,49,50,51,52,55].

## 7. Future Perspectives

SARS-CoV-2 is continuously evolving and showing different characteristics in its transmissibility, infectivity, and immune escape strategies due to mutational changes during replication. Thus far, several variants of concern (VOCs) have been identified, including B.1.1.7 (Alpha), B.1.351 (Beta), P.1 (Gamma), B.1.617.2 (Delta), and B.1.1.529 (Omicron). The Omicron variant is highly virulent and contagious as it accumulates about 50 mutations across its genome, 32 of which are present in the spike protein [56].

Recent reports highlighted the virulence of the Delta variant in severe placentitis and significant fetal injury or distress cases [17,57,58]. Intervillositis increased fibrin deposition and syncytiotrophoblast necrosis along with apoptosis, senescence, and ferroptosis [59]. Another report from the CDC indicated a higher number of stillbirths and severe placentitis cases with the Delta variant [60]. A study from the United Kingdom showed that placentas in asymptomatic women were similar to those of women with severe COVID cases and other comorbidities such as diabetes or obesity [61].

According to the Amsterdam Placental Workshop Group Consensus Statement on Sampling and Definitions, placentitis is universally used to describe the most common pathologic lesions found in the placenta [62]. Placental inflammatory histopathological features are considered similar to those of other pregnancy-related viral RNA infections [63]. Vascular anomalies in both placental and fetal contexts require further investigation due to a lack of significant reports caused by the presence of comorbidities such as chronic gestational hypertension, systemic lupus erythematosus, inherited thrombophilia, coagulation disorders, and/or other maternal conditions in the conducted studies. Investigating the feto-placental unit for SARS-CoV-2 infection could be crucial to understand the probability of in utero programming for young and adult disease cases [64].

Smithgall et al. [65] recently investigated the effects of SARS-CoV-2 messenger RNA (mRNA) vaccination on placental pathology and found no comparable changes between vaccinated and unvaccinated pregnant women, further emphasizing the safety of vaccination for pregnant women [65]. Future studies should also evaluate other factors that may influence placental responses to infection such as ACE2 and the expression of other entry factors; maternal clinical characteristics such as nutritional status, parity, and previous infections with tropical viruses; comorbidities such as pre-eclampsia; and effects on timing and the use of different therapeutic interventions on fetal–maternal outcomes.

Table 2 lists the main factors related to SARS-CoV-2 infection and the feto–placental unit.

## 8. Conclusions

The cumulative published data on placenta with SARS-CoV-2 infection showed common histological features, including vascular malperfusion (MVM and FVM), inflammation, thrombosis, and fibrin deposition. It is crucial to continue research into SARS-CoV-2 infection’s impact on the placenta because unfavorable and extreme obstetric outcomes can occur, albeit rarely; however, available evidence does not identify a strong and absolute correlation between placental lesions, maternal infection, and obstetric outcomes. Further studies are needed to better understand the significance of the timing of maternal SARS-CoV-2 infection, the reality of placental damage, the significance of emerging SARS-CoV-2 variants, the rate of vertical transmission associated with this pattern of placental inflammation, and the role of vaccination. Looking to the future, it is also important to determine how exposure to viral infection by SARS-CoV-2 during pregnancy may affect newborns, children, and/or adults. This research will require long-term follow-up programs.

## Figures and Tables

**Table 1:** Entry factors, other factors, and immunoplacental barriers for SARS-CoV-2 [10,25,26,27,28,29,30,31,32,33,34,35,36,37,38,39,40].

“Entry Factors”	Function/Role
Angiotensin-converting enzyme-2 (ACE2)	The target receptor for SARS-CoV-2.
Trans-membrane serine protease 2 (TMPRSS2), Cathepsin B and L (Cat B/L)	Two host proteases. Enables membrane fusion.
Furin	Reduces the virus’s dependence on proteases for entry
Basigin (BSG/CD147), dipeptidyl peptidase-4 (DPP4/CD26), proteases (elastase, tyrosine, and membrane-associated serine proteinases (MASPs))	May enhance viral infectivity
Basigin and DPP4 co-expression with interferon-induced transmembrane protein (IFITM1-3), lymphocyte antigen 6E (LY6E)	Reduces the permissibility of the placenta to viral entry
Neurolipin 1 (NRP1)	Alternative receptor for SARS-CoV-2 entry into the placenta
Human leukocyte antigen (HLA)-G (HLA-G)	Causes an increase in the immune-tolerogenic molecule HLA-G, which can act as immune escape mechanism for SARS-CoV-2
Podoplanin (PDPN)	Marker of vasculogenesis. Possible down-regulation was observed in the villous stroma of women infected during the third trimester
Toll-like receptors (TLRs), Internalization receptors (E-Cadherin)	Pattern recognition receptors (PRRs); the receptors can be exploited by pathogens or modulated by inflammatory signals

**Table 2:** Key points on SARS-CoV-2 and placenta.

Key Findings
Relatively low rates of SARS-CoV-2 maternal–fetal transmission were observed, but increased risks for the mother and unfavorable feto-neonatal outcomes exist. We observed risks for the mother (respiratory insufficiency, admission to the ICU, and maternal death) and feto-neonatal outcomes (preterm labor and birth, fetal growth restriction, stillbirth, hypertensive disorders, and pre-eclampsia). Pro-inflammatory and pro-coagulation effects of the infection were observed, including “cytokine storm” and renin-angiotensin system down-regulation via ACE2 receptors. In pregnant women with SARS-CoV-2 infection, a significant proportion of placentas showed histopathologic findings, but mainly non-specific anomalies were observed. Maternal infection seems not to equate to placental infection. Evidence of placental viral infection does not guarantee intrauterine vertical transmission to the fetus. Often subclinical placental changes/disfunctions were observed with no fetal compromise/perinatal compromise. Future studies should be updated according to the following: - Emergent virus variants and epidemiological changes; - Gestational age at infection (stratifying outcomes for the trimester); - Previous anti-SARS-CoV-2 vaccination; - Environmental factors. Studies should continue histological examination of placentas (including morphologic and morphometric analysis, immunohistochemistry studies, immunofluorescence, etc.); dedicated anatomical pathologists in perinatal pathology are also desirable. The effect of young-adult diseases on “in utero programming” due to maternal and feto–placental infection should be further explored (long-term follow-up).

## Data Availability

Not applicable.
